# Improvements in Heart Rate Variability, Baroreflex Sensitivity, and Sleep After Use of Closed-Loop Allostatic Neurotechnology by a Heterogeneous Cohort

**DOI:** 10.3389/fpubh.2018.00116

**Published:** 2018-04-25

**Authors:** Hossam A. Shaltout, Sung W. Lee, Catherine L. Tegeler, Joshua R. Hirsch, Sean L. Simpson, Lee Gerdes, Charles H. Tegeler

**Affiliations:** ^1^Hypertension and Vascular Research Center, Wake Forest School of Medicine, Winston-Salem, NC, United States; ^2^University of Arizona School of Medicine, Phoenix, AZ, United States; ^3^Department of Neurology, Wake Forest School of Medicine, Winston-Salem, NC, United States; ^4^Department of Biostatistical Sciences, Wake Forest School of Medicine, Winston-Salem, NC, United States; ^5^Brain State Technologies, LLC, Scottsdale, AZ, United States

**Keywords:** neurotechnology, allostasis, heart rate variability, acoustic stimulation, baroreflex sensitivity, closed-loop, neural oscillations, HIRREM

## Abstract

**Background:**

Heart rate variability (HRV) is an indicator of dynamic adaptability of the autonomic nervous system. Few interventions target upstream, cerebral cortex components of the heart–brain system for autonomic management. We report changes in HRV and baroreflex sensitivity (BRS), associated with use of a noninvasive, closed-loop, allostatic, computer-guided, acoustic stimulation neurotechnology.

**Methods:**

Over 5 years, 220 subjects with heterogeneous neurological, cardiovascular, and psychophysiological conditions consecutively enrolled in a naturalistic, single-arm study exploring clinical effects associated with use of the neurotechnology. Of those, 202 completed the study protocol and 160 had recordings adequate to analyze HRV and BRS. Mean age was 44.0 (SD 19.4), with 130 women. Participants received a mean of 16.1 (5.2) sessions, over 24.2 days (23.3), with 9.5 (3.8) actual intervention days. Sessions included real-time analysis of brain electrical activity and software algorithm-guided translation of selected frequencies into patterns of acoustic stimulation (audible tones of variable pitch and timing), to facilitate auto-calibration of neural oscillations. Outcomes including 10-min supine, at-rest recordings of blood pressure and heart rate, and inventories for insomnia (ISI) and depression (CES-D or BDI-II), were obtained at baseline and 15.3 (16.7) days after the last session.

**Results:**

Compared to baseline, significant increases (all *p* < 0.001) were observed for measures of HRV across all participants including the mean percentage change for SDNN 24.2% (SE 0.04), and RMSSD, 42.2% (0.08), and BRS [Sequence Up, 55.5% (0.09), Sequence Down, 77.6% (0.23), and Sequence All, 53.7% (0.07)]. Significant improvements were noted in SAP, MAP, and DAP, as well as natural log of HF, and total power. Self-reported ISI was reduced (ISI, −6.4 points, SD 5.6, *p* < 0.001). The proportion reporting clinically significant depressive symptoms reduced from 48.2% at baseline to 22.1% at follow-up. Linear regression showed that rightward asymmetry predicted lower SDNN (*p* = 0.02). Exploratory analysis showed a trend for improved balance of temporal lobe high-frequency amplitudes over the course of initial sessions.

**Conclusion:**

These findings indicate that use of a noninvasive, allostatic, closed-loop neurotechnology appears to have robust potential for public health efforts to support greater flexibility in autonomic cardiovascular regulation, through self-optimization of electrical activity at the level of the brain.

## Introduction

Numerous studies have shown that heart rate variability (HRV) is a useful physiological indicator of dynamic adaptability in the autonomic nervous system. In adults, low HRV is a risk factor for adverse cardiovascular outcomes (1, 2), new onset of diabetes (3), progression of chronic kidney disease (4), and all-cause mortality (5). The ubiquity of diminished HRV in behavioral health disorders has led to its proposal as a transdiagnostic biomarker for psychopathology (6). As a measure that can be obtained easily and noninvasively, HRV merits serious consideration as a target for observation and intervention on a public health basis (7). Furthermore, attention to HRV may support the progress of advanced practices which are beneficial for both physical and mental health or adaptive neurovisceral integration (8).

A wide variety of behavioral, physical exercise, and pharmacological therapies have been shown to increase HRV (9). Especially for interventions that entail relatively non-specific features, it seems likely that effects will depend on the capacity to influence both central and peripheral nervous system pathways. An intriguing question is whether focused engagement of critical central structures, especially those known to have specific roles for autonomic management, may be a way to produce more efficient or pronounced effects on HRV. For example, the bihemispheric autonomic model for management of traumatic stress (BHAM) begins with recognition that the right and left hemispheres are primarily responsible for cortical management of the sympathetic and parasympathetic divisions, respectively (10). The BHAM suggests that temporal lobe electrical asymmetry may be an indication of traumatic stress exposure, associated with health effects including reduced HRV, and the model proposes that intervention to reduce asymmetrical activity may be a way to facilitate a state of enhanced autonomic regulation, including increased HRV.

High-resolution, relational, resonance-based, electroencephalic mirroring (HIRREM^®^, Brain State Technologies, Scottsdale, AZ, USA), is a noninvasive, closed-loop, allostatic, acoustic stimulation neurotechnology (11), that is designed to facilitate auto-calibration of neural oscillations. The HIRREM brainwave mirroring interventional strategy aims to facilitate more adaptive forms of symmetry at the temporal lobes and other cortical regions. HIRREM is aligned with the BHAM as well as the broader physiological paradigm of allostasis (stability through change), which recognizes the brain as the organ of the central command (12). As a closed-loop neurotechnology (i.e., an intervention whose inputs are objectively measured real-time neurological data), HIRREM is not intended to depend on conscious, cognitive activity, volitional self-regulation, or behavioral monitoring.

The primary objective of this report is to summarize changes in measures of HRV and baroreflex sensitivity (BRS), as well as self-reported symptoms of insomnia (ISI) and depression, in a large, consecutively enrolled, heterogeneous population of subjects who undertook use of HIRREM. Subsets of these data have been presented earlier for patients with menopausal hot flashes (13), postural orthostatic tachycardia syndrome (14), sport-related concussion (15), and post-traumatic stress (16). The secondary objective is to explore the potential role of temporal lobe high frequency patterns of change in temporal lobe asymmetry that were expressed over the course of the initial five sessions of HIRREM.

## Materials and Methods

### Population and Subject Recruitment

Participants were drawn from among those enrolled between 07/16/2012 and 08/05/2016, in a single site, IRB-approved, open label exploratory study to evaluate the feasibility and effects of HIRREM for individuals with one or more diverse neurological, cardiovascular, or psychophysiological conditions (ClinicalTrials.gov NCT02709369). The study was carried out in the Department of Neurology at the Wake Forest School of Medicine, Winston-Salem, NC, USA. Participants were identified by clinician referral or informal networks, and all provided informed consent. Those unable to provide informed consent, attend study visits, or sit comfortably in a chair were excluded, as were those with bilateral total hearing loss, known seizure disorders, or ongoing use of benzodiazepines, opiate, or anti-psychotic medications.

Following informed consent, participants completed a set of outcome measures (below) before beginning their series of in-office HIRREM sessions, which were all conducted at the clinical study site (details below). Post-intervention outcome measures were repeated at a time following completion of the HIRREM sessions that was convenient for the subject, preferably within 2 weeks of the last session.

### Assessment of HRV and BRS

Continuous recordings of blood pressure (BP) and heart rate (HR) were acquired from noninvasive finger arterial pressure measurements and electrocardiogram for a minimum of 10-min in subjects lying down quietly, supine, breathing freely. Recordings were obtained at the enrollment visit, approximately 30 min before the HIRREM assessment, and at the follow-up visit after completion of the HIRREM intervention. Systolic, diastolic, and mean arterial BP, as well as beat-to-beat RR interval files generated *via* the data acquisition system (BIOPAC acquisition system and Acknowledge 4.2 software, Santa Barbara, CA, USA) at 1,000 Hz were analyzed using Nevrokard SA-BRS software (by Nevrokard Kiauta, d.o.o., Izola, Slovenia). Evaluation included measures of BRS including Sequence UP, DOWN, and ALL, and HRV in both the time and frequency domains. All recordings were visually inspected, and the first 5 min of usable tracings were analyzed using Nevrokard Software to identify R waves from the ECG and BP tracing followed by subsequent determination of HRV and BRS in both time and frequency domains. The primary frequency domain variables of interest were low-frequency (LF) power, and high-frequency (HF) power, the ratio of LF to HF (LF/HF), and the total power determined from power spectral analysis using fast Fourier transformations (Hamming window) with band widths 0.04–0.15 and 0.15–0.40 Hz for LF and HF, respectively. The HF is believed to reflect parasympathetic modulation of HR. And the LF and the LF/HF are commonly used to reflect sympathetic regulation and sympathovagal balance, respectively. The primary time domain variables of interest were the SD of all R to R intervals, commonly reported as N to N intervals (SDNN), the square root of the mean of sum of squares of differences in successive N to N intervals (rMSSD). For all of these variables, a higher value suggests greater HRV. Recordings with dropped beats or gross motion artifact were excluded from analysis.

### Self-Report Measures for Symptoms of ISI and Depressive Mood

The ISI is a 7-item survey that assesses the severity, nature, and impact of ISI symptoms on quality of life over the previous 2 weeks (17). It is scored on a 5-point Likert scale from 0 (no problem) to 4 (very severe problem) on a composite score range from 0 to 28. Composite scores can be stratified into the following clinical severities of ISI: absence (0–7), sub-threshold (8–14), moderate (15–21), and severe (22–28) (18). The ISI’s internal consistency was found to be 0.74 and a correlation with sleep diaries was also established. Depressive mood was measured by the CESD (19) and the BDI-II (20), over the period of the study. Severity of depressive symptomatology was measured dichotomously, using scores of 16 or greater for the CES-D and 14 or greater for the BDI-II.

### HIRREM Intervention

Process and procedures for provision of HIRREM have been discussed in detail previously (11). The initial brainwave assessment consisted of two-channel recordings of brain electrical activity from at least six paired locations on the scalp (F3/F4, C3/C4, T3/T4, P3/P4, FZ/OZ, and O1/O2), with the recipient at rest and while carrying out a task, using sensors and amplifiers (Brain State Technologies, Scottsdale, AZ, USA) that sample at 256 Hz. At each location, data were recorded for 1 min each with eyes closed, eyes partially open as a transition in state of arousal, and eyes open while engaging with a mental task (e.g., reading numbers, performing mental calculations, etc.). Trained technologists evaluated assessment data to choose protocols for the initial HIRREM session.

Intervention protocols included recording brain electrical activity through generally two channels, with scalp sensors placed at homologous regions of the hemispheres according to the 10–20 International EEG system. In real-time, software algorithms analyzed specific ranges of the brain electrical frequency spectrum, identified dominant frequencies on the basis of proprietary mathematical formulae, and translated those frequencies to acoustic stimuli (audible tones of variable pitch and timing). The tones were presented to participants through standard earphones (Creative EP-630 or Sony Stereo Headphones MDR-EX58V) with as little as an 8-ms delay. Volume (decibels) of acoustic stimulation was adjusted by each participant in accordance with their preference.

The HIRREM sessions were scheduled to maximize frequency and efficiency with participants generally completing two sessions in a half day, separated by a break of 20–30 min. Each HIRREM session (approximately 90 min each) consisted of 3–10 HIRREM protocols addressing different locations (3–40 min each), some done with eyes closed and some with eyes open, with the participant being asked to relax while sitting or reclining comfortably in a zero-gravity chair. Specific protocols for successive HIRREM sessions were chosen based on brain electrical data from the preceding session, which for purposes of technologist review was aggregated in broad-band frequency ranges (<1.0, 1.0–3.0, 3.0–5.5, 5.5–7.5, 7.5–10.0, 10.0–12.0, 12.0–15.0, 15.0–23.0, 23.0–36.0, 36.0–48.0 Hz). Special attention was given to activity set points suggestive of dominant hemispheric asymmetries and/or suboptimal ratios of energy across the frequency spectrum. Algorithms are designed to support de-establishment of relatively invariant and potentially maladaptive activity patterns. The decision for the total number of sessions to be received was based on impressions of clinical improvement or plateau, including evaluation of the participants’ brain pattern evolution over the course of their sessions, as well as the participants’ schedules and preferences. All participants continued with their usual medical or behavioral care.

### Main Statistical Analyses

All pre- to post-intervention comparisons for autonomic and self-report measures were conducted with Excel, using paired *t*-tests. Variability estimates for time domain measures of HRV, BRS, BP, and self-report inventories were generated as SDs or SEs. Spectral measures of HRV (LF, HF, and total power) were evaluated as natural logarithms.

### Exploratory Analyses of Temporal Lobe Electrical Asymmetry

A HF (23–36 Hz) band was selected and filtered as the range of interest for analysis on the basis that activity in this range may be taken as an indication of cortical activation (21). Electrical amplitudes (microvolts) in this range were aggregated as a HF band average. The HIRREM approach is designed to be insensitive to recording artifacts (11). To be consistent with the procedural needs of a point-of-care intervention in a resource-sensitive context, no attempt was made to identify sub-epochs of data that may have reflected noncortical factors (e.g., eye blinks or muscular contractions). A temporal lobe HF electrical asymmetry percentage score (eyes closed) was calculated for each subject by subtracting the value for the HF band average at T3 from the value at T4 and dividing by the lesser of the two, yielding a positive score for rightward (T4) asymmetry. Scalp-measured temporal lobe electrical asymmetry has been proposed as a way to assess autonomic tendencies (22) because temporal regions have relative proximity to the insular cortices, which show a division of labor for management of the autonomic nervous system (23, 24).

Evaluation for a potential relationship between temporal lobe HF asymmetry and SDNN was conducted through a linear regression that tested whether baseline HF asymmetry was a predictor of SDNN. An additional model was tested that included age, gender, current beta-blocker usage, and the revised Charlson comorbidity score (25) as covariates.

Evaluation of change in asymmetry over the first five HIRREM sessions at the bilateral temporal lobes was conducted through an exploratory analysis based on the slope of fitted trend lines for changing asymmetry scores at the start of those successive sessions. Subjects were categorized based on their initial temporal lobe dominance shown during their assessment (eyes closed). Rightward dominance was defined as temporal lobe HF asymmetry of 10% or greater; leftward dominance was defined as asymmetry of −10% or lesser; and symmetry was defined as between −10 and 10%. Except for the first 15-s epoch, data from the first 7 min for each of the first five sessions were analyzed to produce 27 serial asymmetry scores per session (15 s per epoch), which were averaged across all subjects for each dominance group. To assess whether the fitted lines reflected a tendency for change in asymmetry score, a mixed model *F*-test was performed on each slope (SAS, Cary, NC, USA) to account for within-subject temporal correlation, with the null hypotheses being that the slope was 0.

## Results

**Figure 1:**
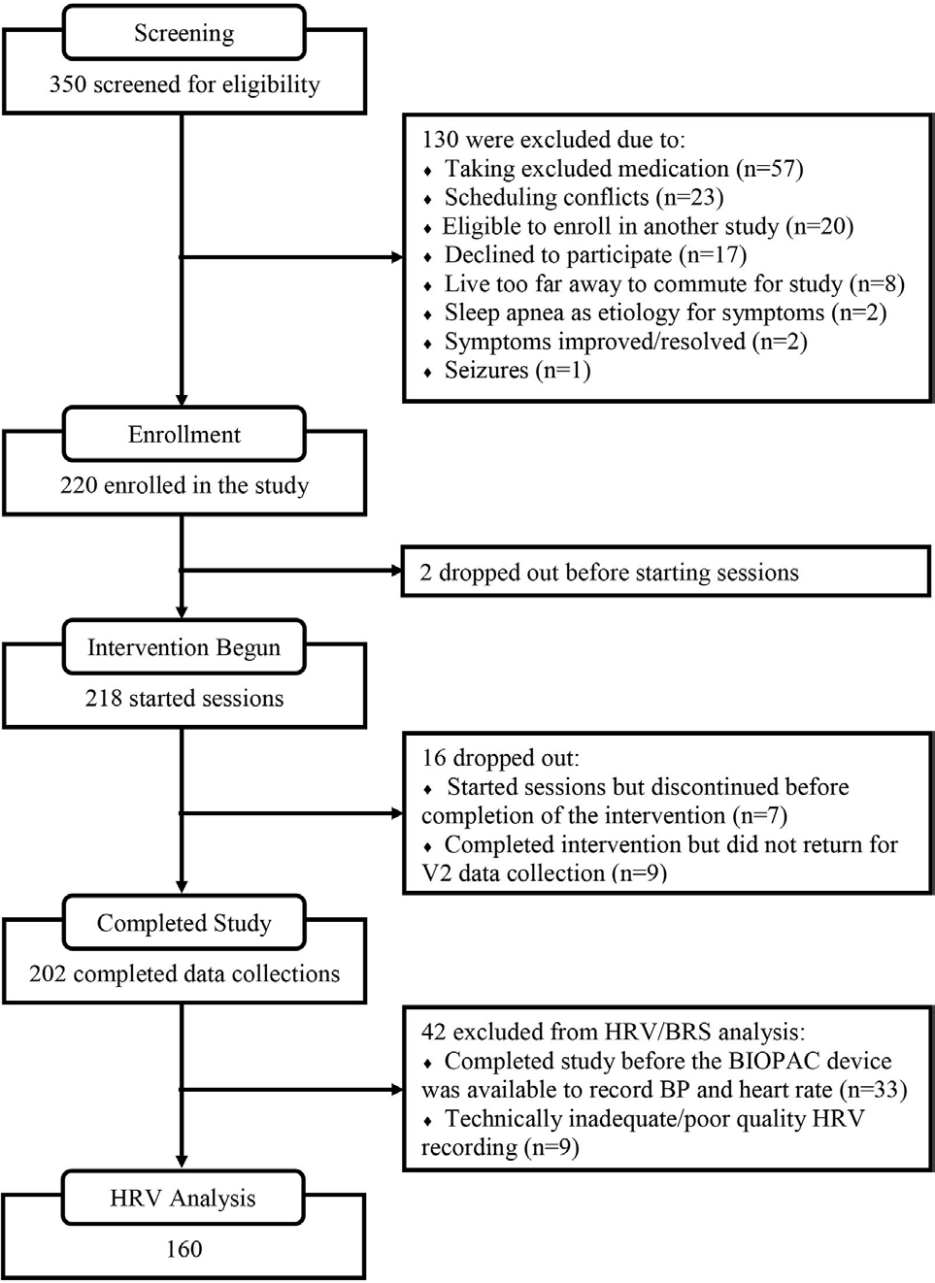
Study participant flow.

A summary of the flow for participant recruitment, screening, enrollment, intervention usage, and follow-up is shown in Figure 1. At the screening stage, the need for ongoing usage of a benzodiazepine, opioid, or anti-psychotic medication was the most common reason for ineligibility. Sixty-four percent of the enrolled subjects were women, mean age was 44.0 (SD 19.4, range 13–83). Mean score on the Charlson comorbidity index was 0.9 (SD 1.1, range 0–8), and Table 1 provides a listing of comorbid health conditions that subjects reported. Clinical diagnoses which subjects gave as their primary motivations for enrolling in the study were ISI (26.7%), traumatic brain injury or concussion (15.8%), menopausal hot flashes (8.9%), post-traumatic stress (8.9%), migraine or headache (7.4%), postural orthostatic tachycardia syndrome (5.0%), or other conditions including anxiety or depression, fatigue or burnout, cancer recovery, autonomic or neurological disorders, and others (27.2%). Participants received a mean of 16.1 (SD 5.2) HIRREM sessions, and there was a mean of 15.3 (16.47) days between the last session and the follow-up visit. There were no serious adverse events reported.

**Table 1:** Self-reported health conditions of study participants.

Condition	Number of participants (percent of *n* = 202)
ADD/ADHD	28 (13.86)
Chronic fatigue	22 (10.89)
Chronic pain	32 (15.84)
Concussion/traumatic brain injury	62 (30.69)
Depression	74 (36.63)
Headaches	84 (41.58)
Hot flashes	44 (21.78)
Hyperlipidemia	25 (12.38)
Hypertension	38 (18.81)
Insomnia	87 (43.07)
Migraines	65 (32.18)
PTSD	29 (14.36)
Stress/anxiety	84 (41.58)

**Figure 2:**
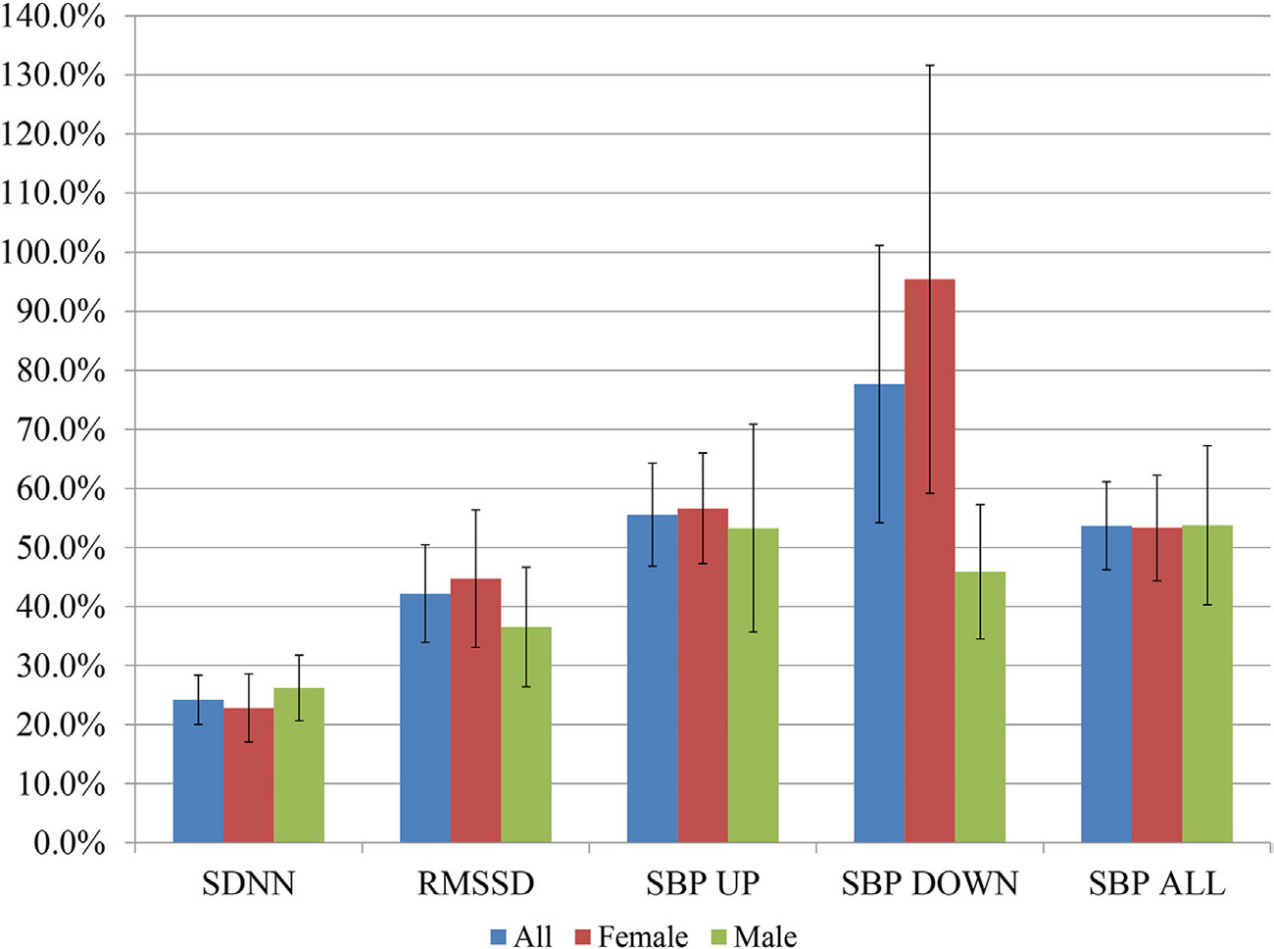
Post-interventional changes in heart rate variability and baroreflex sensitivity on percentage basis. Bars indicate ± SEs for the average percent changes. All changes were statistically significant at *p* < 0.001 except the following, for men: RMSSD, SBP DOWN, SBP ALL (*p* < 0.01 for each), and SBP UP (*p* = 0.02).

**Figure 3:**
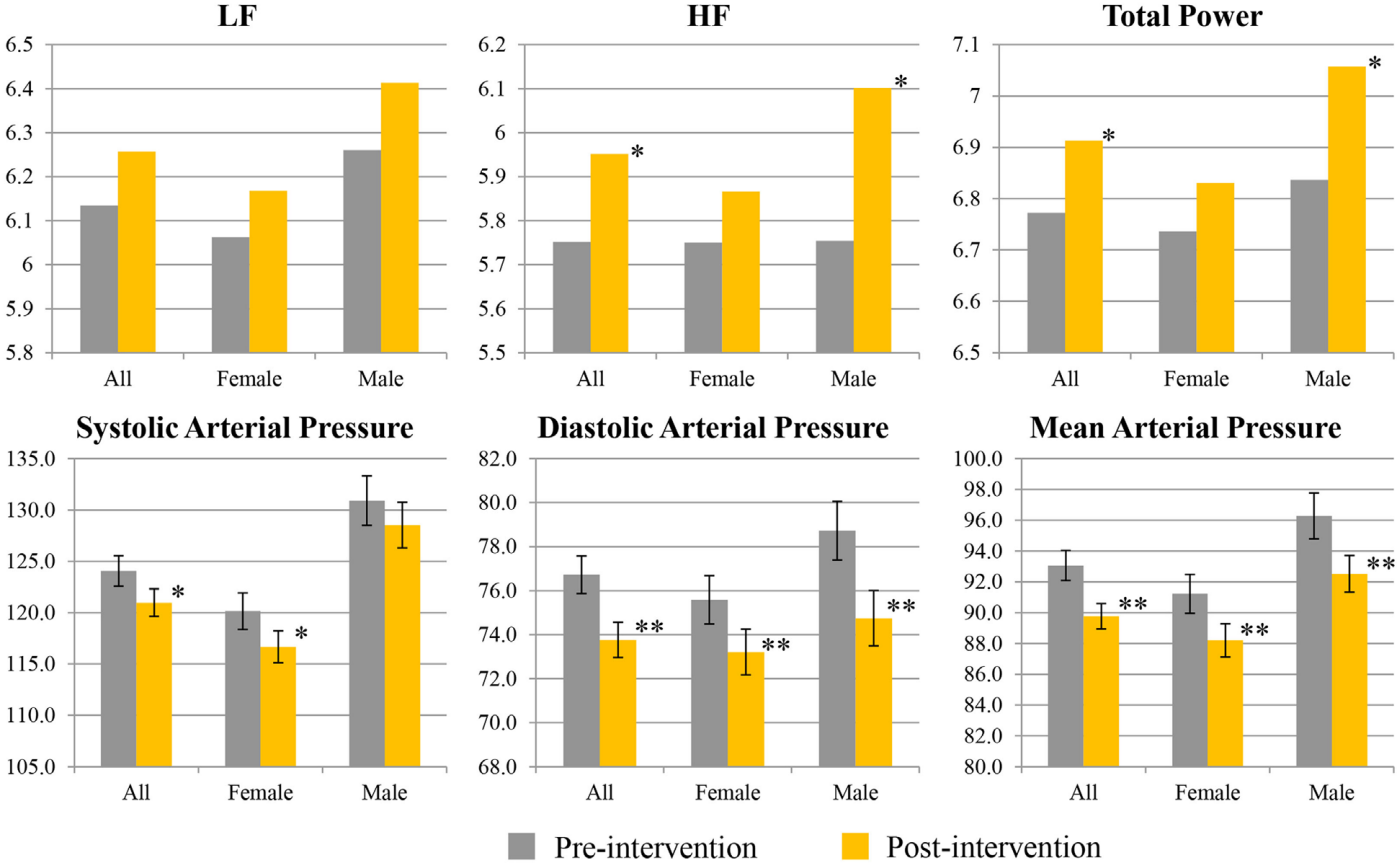
Pre- and post-interventional values for spectral measures of heart rate variability, and blood pressure. Values for low frequency (LF), high frequency (HF), and total power are given as natural logarithms of the absolute values. Values for systolic, diastolic, and mean arterial pressure are given as millimeters of mercury. Single asterisk indicates 0.1 ≤ *p* ≤ 0.05; double asterisks indicate *p* < 0.01.

**Figure 4:**
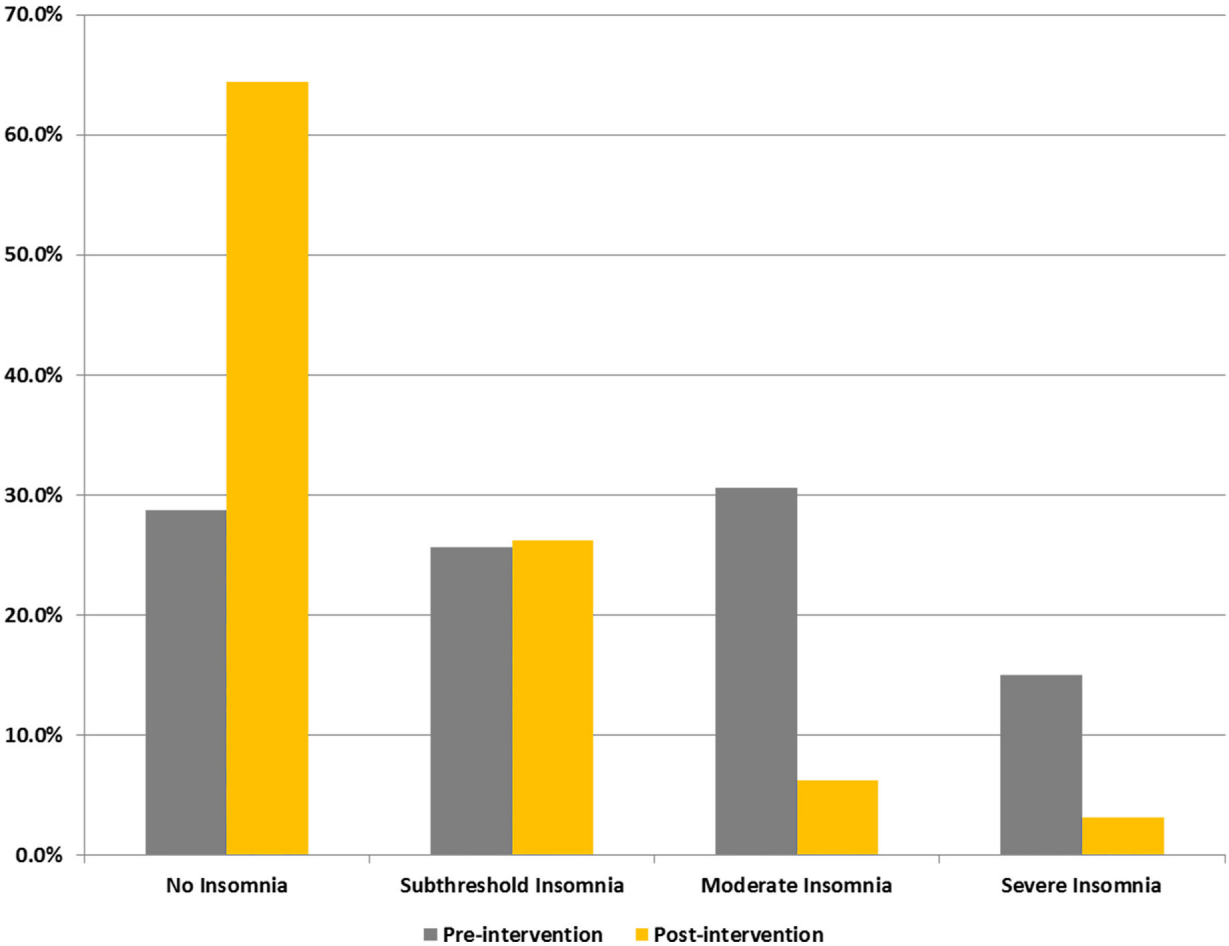
Proportions of subjects reporting different levels of insomnia (ISI) symptom severity. Categorizations are based on self-reported scores on ISI.

Figure 2 shows percentage changes for measures of HRV in the time domain and BRS, and Figure 3 shows pre- and post-intervention values for frequency domain HRV measures and BP, before and after usage of HIRREM (*n* = 160). This included significant changes in measures of HRV (*p* < 0.001 for these measures) across all participants including the mean percentage change for SDNN 24.2% (SE 0.04), and RMSSD, 42.2% (0.08), and BRS [Sequence Up, 55.5% (0.09), Sequence Down, 77.6% (0.23), and Sequence All, 53.7% (0.07)]. Significant improvements were also noted in SAP, MAP, and DAP, as well as natural log of HF, and total power. The baseline value for SDNN across all subjects was 44.1 (SD 20.8), and it was 52.0 (SD 25.9) at the follow-up visit. Pre- and post-intervention values for the measures when participants were stratified according to their baseline value for SDNN and LF power are shown in Tables 2 and 3, respectively. On average, all baseline SDNN quartiles showed improvements in SDNN and BRS. Those in the lowest quartile for LF power showed an increase in their mean LF power, and those in the highest group showed a decrease. Mean change in the ISI was −6.4 points (SD 5.6, *p* < 0.001), and the proportion of subjects in different clinical categories of ISI symptom severity before and after the intervention are shown in Figure 4. At baseline, 48.2% of subjects reported clinically significant levels of depressive mood, while 22.1% did so at follow-up.

**Table 2:** Heart rate variability, baroreflex sensitivity, and blood pressure changes by quartile of SDNN.

	Quartile 1		Quartile 2		Quartile 3		Quartile 4	
	V1	V2	V1	V2	V1	V2	V1	V2
Ln LF	4.6	4.9	5.8	6.0	6.5	6.7	7.6	7.4
Ln HF	4.1	4.7	5.6	5.6	6.1	6.2	7.2	7.3
Ln TP	5.2	5.6	6.5	6.6	7.1	7.2	8.3	8.2
UP SBP	6.7 (4.4)	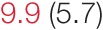	11.9 (5.6)	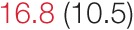	15.0 (7.8)	19.2 (12.3)	28.6 (13.8)	32.6 (16.1)
DOWN SBP	7.3 (3.4)	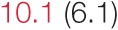	12.9 (7.4)	16.1 (8.5)	14.2 (6.8)	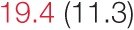	23.4 (9.7)	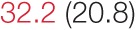
ALL SBP	6.5 (2.8)	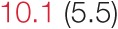	12.2 (5.3)	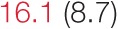	14.4 (6.6)	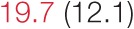	26.1 (10.2)	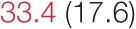
SDNN	21.6 (5.8)	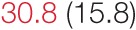	35.7 (3.3)	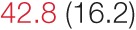	46.6 (4.3)	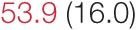	73.5 (14.8)	80.6 (24.0)
RMSSD	12.9 (5.0)	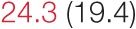	26.2 (9.4)	31.3 (17.0)	37.0 (13.8)	42.3 (18.6)	65.6 (21.9)	74.2 (30.0)
Heart rate	72.8 (11.0)	70.8 (9.8)	72.1 (17.8)	71.3 (11.0)	67.2 (8.3)	68.2 (10.6)	61.0 (9.8)	60.6 (9.3)
SAP	128.5 (18.4)	124.9 (15.6)	122.9 (18.4)	117.0 (16.8)	127.1 (19.2)	125.1 (18.4)	117.7 (18.0)	116.9 (15.6)
MAP	96.7 (11.4)	93.2 (10.2)	92.1 (11.6)	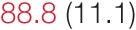	94.9 (11.7)	90.9 (10.4)	88.5 (13.7)	86.2 (8.9)
DAP	79.5 (9.0)	76.2 (8.7)	76.2 (10.4)	74.5 (10.7)	77.7 (10.1)	74.0 (11.0)	73.6 (12.8)	70.4 (9.4)

*Pre- and post-intervention values are shown for subjects with lower (quartiles 1 and 2) and higher (quartiles 3 and 4) baseline levels of SDNN. Low frequency, high frequency, and total power values are shown as natural logarithms of the absolute power values. SDs are indicated in parentheses. Statistically significant differences between pre- and post-interventional values are shown with colored font (blue for 0.01 ≤ *p* ≤ 0.05; red for *p* < 0.01)*.

**Table 3:** Heart rate variability (HRV), baroreflex sensitivity, and blood pressure changes by quartile of low-frequency (LF) power.

	Quartile 1		Quartile 2		Quartile 3		Quartile 4	
	V1	V2	V1	V2	V1	V2	V1	V2
Ln LF	4.3	5.0	5.8	6.0	6.6	6.6	7.8	7.5
Ln HF	4.3	4.9	5.4	5.5	6.2	6.2	7.1	7.3
Ln TP	5.2	5.7	6.4	6.5	7.2	7.2	8.3	8.2
UP SBP	8.2 (5.6)	10.8 (7.1)	10.9 (6.5)	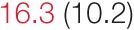	17.3 (9.1)	20.0 (14.8)	26.0 (14.8)	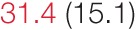
DOWN SBP	8.5 (4.7)	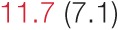	11.6 (6.6)	15.7 (8.8)	16.2 (8.9)	18.8 (14.0)	21.6 (10.0)	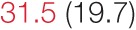
ALL SBP	7.9 (4.5)	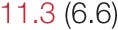	11.1 (5.7)	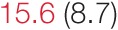	16.4 (8.0)	20.2 (14.3)	24.0 (11.2)	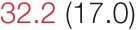
SDNN	24.7 (9.9)	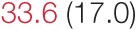	36.2 (7.9)	42.4 (17.0)	47.0 (13.3)	52.3 (21.2)	69.4 (17.8)	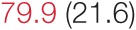
RMSSD	18.1 (12.5)	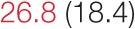	24.6 (11.7)	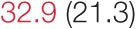	37.3 (18.2)	40.9 (21.8)	61.8 (23.7)	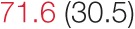
Heart rate	72.2 (17.5)	69.6 (9.4)	70.9 (11.1)	70.7 (11.6)	70.7 (9.2)	71.3 (10.4)	59.3 (8.4)	59.3 (7.8)
SAP	127.8 (18.5)	125.8 (15.2)	126.3 (19.7)	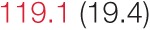	122.1 (16.4)	119.7 (15.4)	120.0 (20.0)	119.2 (17.3)
MAP	95.3 (18.5)	92.9 (10.5)	94.9 (11.7)	89.9 (12.0)	91.6 (12.3)	88.9 (10.2)	90.4 (13.4)	87.4 (8.4)
DAP	78.0 (9.9)	75.4 (9.9)	77.9 (9.7)	75.1 (11.1)	76.1 (11.5)	73.3 (10.5)	75.0 (11.9)	71.3 (8.9)

*Pre- and post-intervention values are shown for subjects with lower (quartiles 1 and 2) and higher (quartiles 3 and 4) baseline levels of LF HRV power. LF, high frequency, and total power values are shown as natural logarithms of the absolute power values. SDs are indicated in parentheses. Statistically significant differences between pre- and post-interventional values are shown with colored font (blue for 0.01 ≤ *p* ≤ 0.05; red for *p* < 0.01)*.

**Figure 5:**
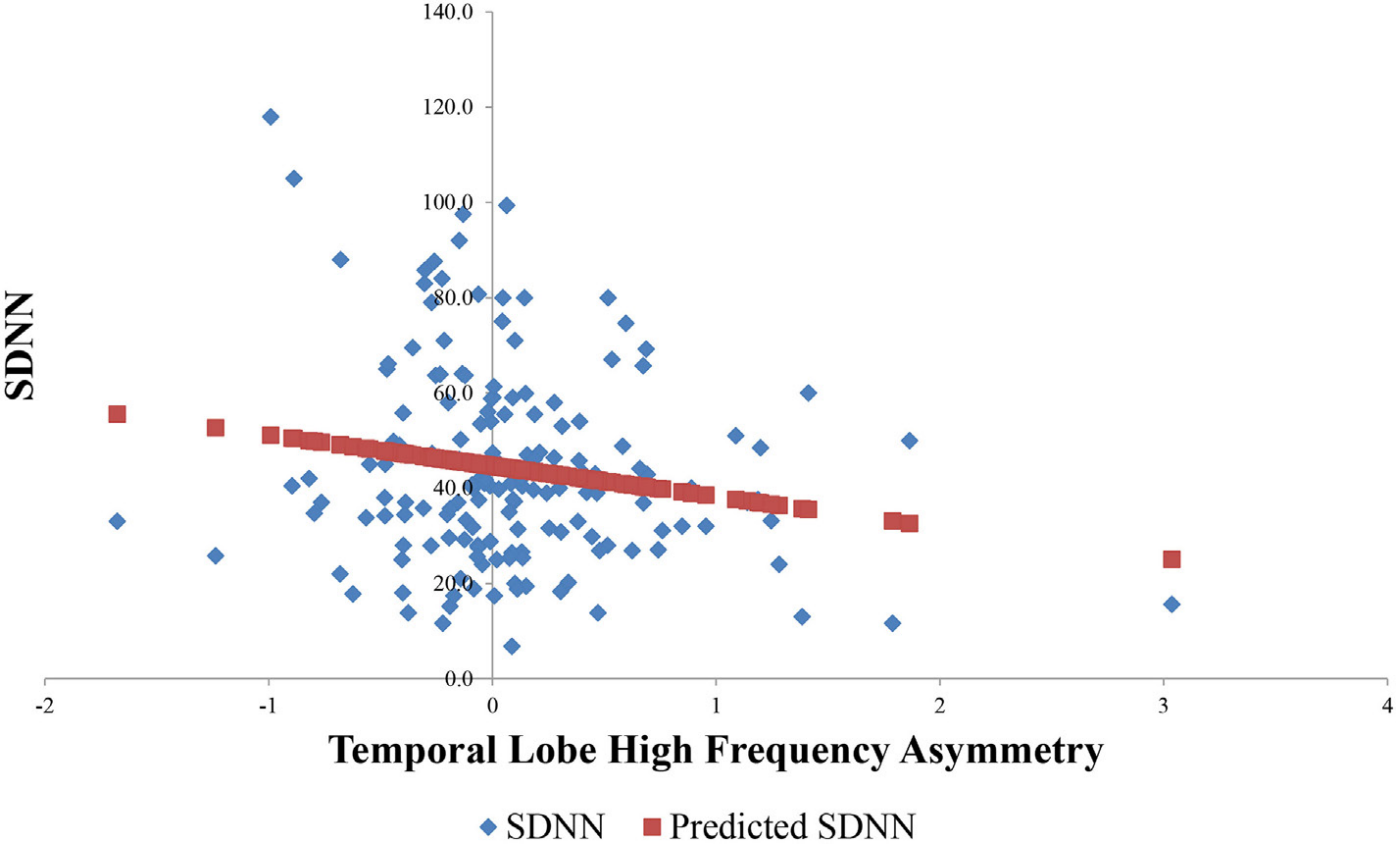
Relationship between baseline temporal lobe high frequency (HF) asymmetry and SDNN. Horizontal axis indicates temporal lobe HF asymmetry score, with positive scores indicating rightward asymmetry and negative scores indicating leftward asymmetry.

**Figure 6:**
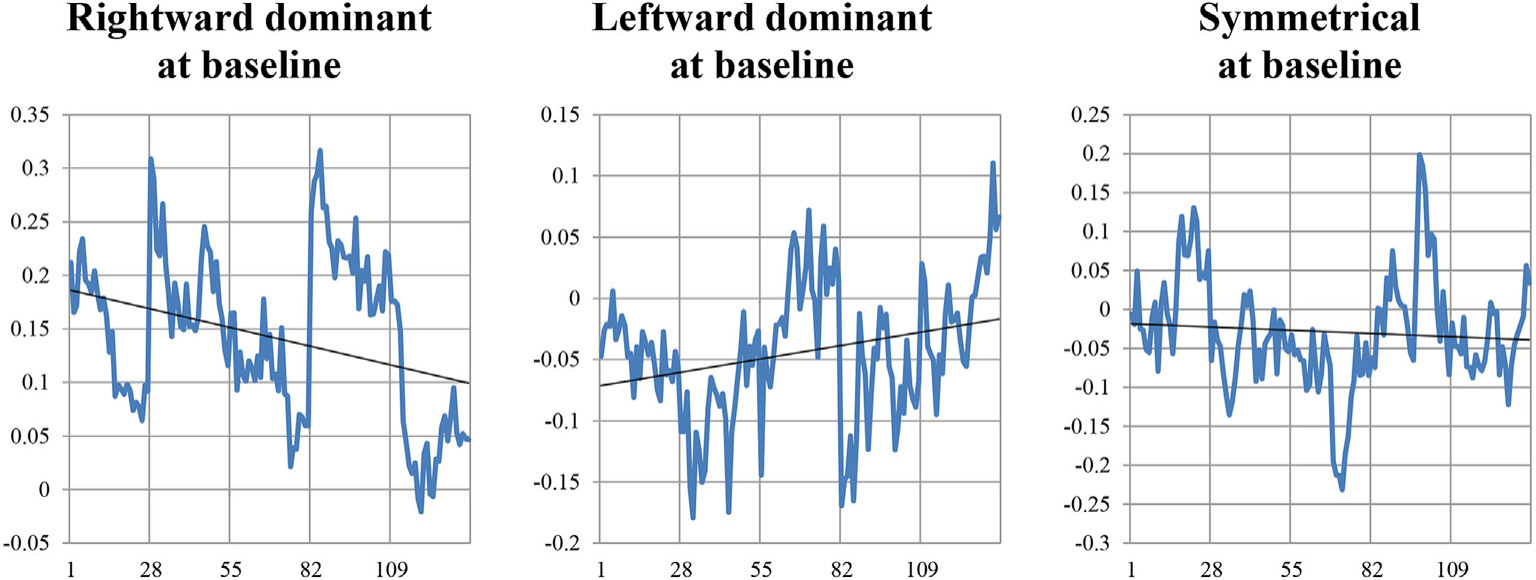
Temporal lobe high frequency asymmetry scores during the initial five HIRREM sessions. Vertical axes indicate values for asymmetry scores calculated from serial 15-s epochs (27 per session), from the first 7 min of a temporal lobe exercise conducted during that session. Gridlines along horizontal axis indicate the start of each session.

Result of a linear regression to explore for the contribution of temporal lobe HF electrical asymmetry toward SDNN is shown in Figure 5. Rightward asymmetry was a predictor of lower SDNN (β coefficient = −6.5, *p* = 0.023). In a model for SDNN that included age, gender, beta-blocker usage, and Charlson comorbidity score as covariates, the relationship between temporal lobe HF asymmetry was increased (β = −8.1, *p* = 0.002). Figure 6 shows the average temporal lobe HF asymmetry values for the first 7 min of each of the first five HIRREM exercises at the temporal lobes (concatenated), when subjects were categorized according to their asymmetry status as measured during the baseline assessment. For subjects who were rightward or leftward dominant at baseline, the slopes of their trend lines for their asymmetry scores over those sessions were negative and positive, respectively; however, analysis did not indicate a statistically significant likelihood of these slopes being non-zero.

## Discussion

This report summarizes main findings from an ongoing prospective, single-arm study involving usage of a closed-loop, allostatic neurotechnology. At a follow-up visit that occurred on average just over 2 weeks after intervention completion, individuals with heterogeneous clinical conditions showed statistically significant increases in HRV and BRS; decreases in systolic, diastolic, and mean arterial pressure; and reductions in symptoms of ISI and depression. The results appear to indicate that significant impact on HRV and related measures of autonomic cardiovascular regulation, as well as improvements in sleep and mood, are possible through use of a well-tolerated, noninvasive, and non-pharmacological intervention for auto-calibration of neural oscillations. The expression of these changes in a heterogeneous cohort is encouraging for the prospect of impacting public health without targeting specific clinical diagnoses.

In broad terms, the HIRREM approach is aligned with other closed-loop interventional strategies that are intended as major advances for neurological and psychiatric disorders, sleep enhancement, and potentially for performance optimization (26–29). Closed-loop neurotechnologies leverage real-time analysis of biological functioning to permit direct, precision-guided modulation of the neural substrates of mentation, emotion, or behavior. As components of a potential public health agenda, noninvasive closed-loop interventions may hold special promise if they are shown to be safe, cost-effective, acceptable, and scalable. Recently, a self-care configuration (Braintellect-2^®^; Brain State Technologies, Scottsdale, AZ, USA) of the closed-loop allostatic neurotechnology evaluated in this study has been developed with support from the United States Army Research Office (30) with sensor placements for temporal and prefrontal cortices only, and integration of this device is envisioned to improve the cost-effectiveness and scalability of neurotechnology-based auto-calibration of neural oscillations.

Given its naturalistic character and in the absence of a control arm, this study was not intended to permit definitive inferences about the specific etiology for changes observed post-intervention. Nonetheless, the relative magnitude and time frame of the HRV increases are noteworthy even if the outcomes were due to non-specific factors including subjective expectation, social interactions with study personnel, or other components of the placebo effect. For the group as a whole, the increase in SDNN compares favorably to the average improvement of 15.9% that was reported in a meta-analysis of interventions to improve HRV (9). The time interval between the last HIRREM session and the follow-up data collection is likely to have been too long for the HRV changes to reflect a short-term state change through temporary relaxation induction. It is also likely to have been too short to reflect HRV change due to an undocumented behavioral cointervention such as aerobic exercise training, which is typically shown to occur after a period of months (31–33).

Our study population was heterogeneous and the inclusion criteria were deliberately transdiagnostic. Analyses based on autonomic cardiovascular regulation profiles revealed findings which may inform future studies of interventions to promote neurovisceral integration. On average, and when stratified according to baseline quartiles of SDNN, all subjects showed greater SDNN after usage of HIRREM. This finding is consistent with the supposition that autonomic regulation is diminished in a wide range of clinical conditions (34), and that potentially beneficial increases in HRV may be achievable regardless of one’s baseline. From the public health perspective, it may be that a campaign for HRV improvement could yield benefits for clinical populations without necessarily targeting those with the lowest HRV. Furthermore, it was intriguing that stratification of subjects by their baseline quartile of LF power showed a post-intervention increase in average power for the lowest quartile, and a decrease in power for the highest quartile. Since LF power comprises both sympathetic and parasympathetic influences (35), this finding gives ground to speculate whether diminished, or high, levels of LF power are a specific reflection of relative activity in one or the other of the autonomic divisions, and whether allostatic neurotechnology can facilitate activity in both divisions to move in healthful directions.

The finding that baseline rightward temporal lobe HF asymmetry was a negative predictor for SDNN is consistent with a cross-sectional analysis we performed previously on a subset of the current subject population (22), which was focused on those individuals with greater degrees of asymmetry. Although the slopes of trend lines for asymmetry did not statistically differ from zero during the first five HIRREM sessions, when comparing individuals who were rightward or leftward dominant at baseline, a similar analysis on a subset of the current group (16) who specifically reported symptoms of post-traumatic stress did show statistical significance. The demographic and clinical heterogeneity of the current sample may have diluted the capacity to detect auto-calibration of neural oscillations, and future studies using larger samples and other analysis strategies may shed additional light on potential mechanisms of HIRREM effects.

## Conclusion

A heterogeneous clinical population undertook usage of a closed-loop, allostatic neurotechnology, HIRREM, for remediation of a wide range of health concerns. Two weeks after concluding their sessions, they demonstrated significant improvements in HRV and BRS, reductions in systolic, diastolic, and mean arterial BP, as well as decreased symptoms of ISI and depressive mood. The intervention was well-tolerated, and there were no adverse events. In aggregate, the findings suggest that closed-loop allostatic neurotechnology could serve as a valuable component of a public health initiative for enhancement of HRV.

## Ethics Statement

This study was carried out in accordance with the recommendations of the Institutional Review Board of Wake Forest University Health Sciences, with written informed consent from all subjects. All subjects gave written informed consent in accordance with the Declaration of Helsinki. Parental written consent was obtained for all participants that were minors. The protocol was approved by the Institutional Review Board of Wake Forest University Health Sciences.

## Author Contributions

Author contributions included conception and study design (CHT, CLT, SL, LG, and HS), data acquisition (CLT, SS and HS), data preparation and analysis (CHT, CLT, HS, SL, HS, SS, and JH), and interpretation of data for this project (CHT, CLT, HS, SL, and LG). All authors have reviewed the manuscript and agreed to be accountable for all aspects of this work.

## Conflict of Interest Statement

CHT, CLT, HS, SS, and JH have no conflicts to report. LG is, and SL was formerly, an employee of Brain State Technologies, Scottsdale, AZ, USA. The reviewer MA declared serving as a consultant on a past grant application with one of the authors CHT to the handling Editor.
